# Auditory guidance of eye movements toward threat-related images in the absence of visual awareness

**DOI:** 10.3389/fnhum.2024.1441915

**Published:** 2024-08-08

**Authors:** Junchao Hu, Stephanie Badde, Petra Vetter

**Affiliations:** ^1^Department of Psychology, University of Fribourg, Fribourg, Switzerland; ^2^Department of Psychology, Tufts University, Medford, MA, United States

**Keywords:** continuous flash suppression, eye movements, sounds, visual awareness, threat, multisensory interaction, superior colliculus, amygdala

## Abstract

The human brain is sensitive to threat-related information even when we are not aware of this information. For example, fearful faces attract gaze in the absence of visual awareness. Moreover, information in different sensory modalities interacts in the absence of awareness, for example, the detection of suppressed visual stimuli is facilitated by simultaneously presented congruent sounds or tactile stimuli. Here, we combined these two lines of research and investigated whether threat-related sounds could facilitate visual processing of threat-related images suppressed from awareness such that they attract eye gaze. We suppressed threat-related images of cars and neutral images of human hands from visual awareness using continuous flash suppression and tracked observers’ eye movements while presenting congruent or incongruent sounds (finger snapping and car engine sounds). Indeed, threat-related car sounds guided the eyes toward suppressed car images, participants looked longer at the hidden car images than at any other part of the display. In contrast, neither congruent nor incongruent sounds had a significant effect on eye responses to suppressed finger images. Overall, our results suggest that only in a danger-related context semantically congruent sounds modulate eye movements to images suppressed from awareness, highlighting the prioritisation of eye responses to threat-related stimuli in the absence of visual awareness.

## Introduction

Detecting threats in the environment is a key survival function that our brain has to fulfill. The human visual system demonstrates a remarkable efficiency in processing signals of potential threat, even when those signals are not consciously perceived (for reviews see Öhman et al., 2007; Tamietto and De Gelder, 2010; Hedger et al., 2015; Diano et al., 2017; Bertini and Làdavas, 2021). For example, subliminally primed images signaling danger evoke heightened social avoidance (Wyer and Calvini, 2011). Angry faces suppressed from awareness modulate likeability ratings of neutral items (Almeida et al., 2013). Suppressed fearful faces evoke larger skin conductance responses (Lapate et al., 2014) and break into awareness faster (Yang et al., 2007; Hedger et al., 2015). Fearful faces located in the blind field of hemianopic patients facilitate discrimination of simple visual stimuli presented in their intact visual field (Bertini et al., 2019). Unaware visual processing of threat-related images, such as fearful faces, is subserved by subcortical structures such as the amygdala (Jiang and He, 2006; Tamietto and De Gelder, 2010; Méndez-Bértolo et al., 2016; Wang et al., 2023), the superior colliculus and pulvinar (LeDoux, 1998; Morris et al., 1998; Whalen et al., 1998; Bertini et al., 2018), and the visual cortices (Pessoa and Adolphs, 2010).

Human oculomotion studies provide insights into how aware and unaware visual stimuli influence actions (e.g., Rothkirch et al., 2012; Glasser and Tadin, 2014; Spering and Carrasco, 2015). Under aware viewing conditions, fearful faces and body postures elicit faster saccadic orienting responses than neutral stimuli (Bannerman et al., 2009). Moreover, oculomotor actions are differentially guided by fearful faces even in the absence of visual awareness. That is, even when fearful faces are entirely suppressed from visual awareness using continuous flash suppression (CFS, Tsuchiya and Koch, 2005), the eyes are attracted toward fearful faces, and repulsed away from angry faces (Vetter et al., 2019). Given the important role of the superior colliculus in controlling eye gaze (Munoz and Everling, 2004) and relaying sensory information (Stein and Meredith, 1993; LeDoux, 1998), it is possible that the superior colliculus, together with the amygdala, mediates the convergence of unconscious threat processing and reflexive oculomotor responses.

In a sensory rich world, threat is conveyed through multiple modalities. Sounds not only arrive faster in the brain than visual signals, threat-related sounds in particular evoke arousal (Burley et al., 2017), capture attention (Zhao et al., 2019; Gerdes et al., 2021), and alert the brain via the brain stem reflex (Juslin and Västfjäll, 2008). The amygdala and the auditory cortex respond interactively to aversive sounds and their perceived unpleasantness (Kumar et al., 2012, for a review, see Frühholz et al., 2016). The primary auditory cortex encodes and predicts threat from fear-conditioned sounds (Staib and Bach, 2018). Further, the superior colliculus is a key mediator for eye responses to sounds, for example, saccadic orienting (see Hu and Vetter, 2024, for a review). However, few studies have tested how threat-related cross-modal stimuli influence eye movements. Responding to threat under natural conditions usually involves sensory processing of concurrent visual and auditory cues. Sounds, in particular, can be heard from all directions and travel around obstacles. Thus, sounds might allow individuals to detect potential threats that are not immediately visible, such as an animal behind a bush. Here, we tested how the eyes respond to threat by using ecologically valid, multimodal stimuli.

Integrating information provided through the different senses improves perception. For example, audiovisual stimulus pairs are detected faster (Miller, 1986) and localized with higher precision (Alais and Burr, 2004) than the same stimuli presented unimodally. Multisensory integration is guided by causal inference: the degree of integration depends on the probability that the signals from the different senses share a common origin (Körding et al., 2007; Badde et al., 2021; Shams and Beierholm, 2022). Accordingly, semantically congruent information in different modalities is more likely to be integrated than incongruent information (Dolan et al., 2001; Doehrmann and Naumer, 2008; Noppeney et al., 2008). Although multisensory integration *per se* requires awareness of the integrated information (Palmer and Ramsey, 2012; Montoya and Badde, 2023), aware information in one modality can facilitate the breakthrough into awareness for suppressed, congruent information presented in another modality (Mudrik et al., 2014). For example, congruent sounds can facilitate the detection and identification of visual stimuli suppressed from awareness (Chen and Spence, 2010; Conrad et al., 2010; Chen et al., 2011; Alsius and Munhall, 2013; Lunghi et al., 2014; Aller et al., 2015; Lee et al., 2015; Delong and Noppeney, 2021) as do congruent haptic stimuli (Lunghi et al., 2010; Hense et al., 2019). This facilitation potentially relies on an established association between the visual and auditory signals (Faivre et al., 2014). Consistently, cross-modal facilitation in the absence of awareness depends on semantic congruency: Chen and Spence (2010) paired backward masked images with naturalistic sounds, including threat-related sounds such as gunshots, helicopter and motor engine sounds. When the sounds were semantically congruent to the masked image, naming the object depicted in the image was facilitated; in turn, naming the object was compromised when sounds were incongruent. Similarly, semantically congruent sounds (car engine or bird singing), compared to irrelevant sounds, prolonged the perceptual dominance of the corresponding image under binocular rivalry conditions (Chen et al., 2011). Interestingly, an overall, sound-independent perceptual dominance of car over bird images emerged in this study. Given that cars can have a threatening character, this dominance of car images ties nicely with previous results showing that threat-related visual images break more easily into awareness.

The present study was designed to probe whether the influence of threat-related images suppressed from awareness on oculomotor actions is modulated by congruent, threat-related sounds. As in a previous study, we combined CFS with eye tracking (Vetter et al., 2019) and measured observers’ eye movements to suppressed images of a car (threat-related) or human hands (not threat-related) while either sounds of a car engine or finger snapping (or no sound) were simultaneously presented. In contrast to previous studies measuring enhanced visual detection or faster break-through of unaware threat-related images into awareness, we tracked the eyes to directly examine the influence of sounds on actions toward suppressed images. By focusing on oculomotor responses, which the participant was not aware of, we controlled for potential confounds (Stein et al., 2011) such as faster button presses in the presence of fear-inducing sounds (Globisch et al., 1999) or fear-inducing images (Bannerman et al., 2009) and thus can directly link changes in eye gaze behavior to changes in visual stimulus awareness.

We chose car stimuli as those have been shown to gain preferential access to awareness just as other fear-related stimuli (Chen et al., 2011) and are associated with threat-related, semantically congruent sounds such as roaring engines. We chose hand stimuli based on evidence showing that the brain distinguishes between human-related stimuli and inanimate stimuli, such as human hands (Bracci et al., 2018) and vehicles (DiCarlo et al., 2012), with specific brain pathways and regions dedicated to the processing of these categories (Caramazza and Shelton, 1998; Kriegeskorte et al., 2008; Grill-Spector and Weiner, 2014; Sha et al., 2015; Bracci and Op De Beeck, 2023). Also, human and inanimate sound categories induce differential representations in visual cortex even in the absence of visual stimulation (Vetter et al., 2014). Thus, given that human hand and inanimate car images are treated distinctively by the brain and have a differential relationship to threat, we predicted that the eyes are guided differentially by these images when they are suppressed from visual awareness. Given that threat-related signals are prioritized in unaware processing, we hypothesized that congruent car sounds may affect eye gaze to unseen car images.

## Materials and methods

### Participants

Twenty participants (17 females, mean age 23.5 years) were included in the final analysis. We determined the sample size based on simulations of our statistical model (see Cary et al., 2024 for an example). Data of three additional participants were excluded from all analyses; for one of them the continuous flash suppression worked only in 37% of trials resulting in sparse eye data (<75%). Two of them performed clearly above chance level (38 and 47% correct responses with chance being at 25%) when indicating the location of the suppressed stimulus despite reporting to not have seen the stimulus. All participants took part in exchange for payment, indicated normal or corrected-to-normal vision and normal hearing, and signed an informed consent form prior to the start of the experiment. The experiment was approved by the Psychology Ethics Committee of the University of Fribourg.

### Apparatus and stimuli

Participants positioned their head on a chin and forehead rest to stabilize head position and viewed a monitor (ROG Strix XG258Q Series Gaming LCD Monitor, 60 Hz, 1,280 × 1,024 resolution) through a mirror stereoscope (ScreenScope, ASC Scientific, Carlsbad, United States). The distance between the stereoscope and the chin rest ranged from 10 to 13 cm; the position of the stereoscope was adjusted for each participant to ensure good stereo fusion while allowing the eye-tracker to catch the eye from underneath the stereoscope. The monitor was placed at a distance of 100 cm from the participant (the target image subtending 1.97° × 2.92° of visual angle).

Twelve static images (six of snapping fingers, six of cars) were selected as visual stimuli. The angle from which the hands and cars were shown varied across images (Supplementary Figure S1). Raw images were processed in Adobe Photoshop (version 23.2.2). The images were first rendered achromatic, the background was removed and replaced with a mid-gray (RGB 128, 128, 128) layer, and the contrast level of the foreground containing the object was reduced. Images were cut to a size of 115 by 173 pixel. All image foregrounds were equated for overall luminance (most frequent luminance in the foregrounds) and contrast (i.e., the standard deviation of the luminance distribution) using the SHINE toolbox (Willenbockel et al., 2010) in MATLAB.

**Figure 1:**
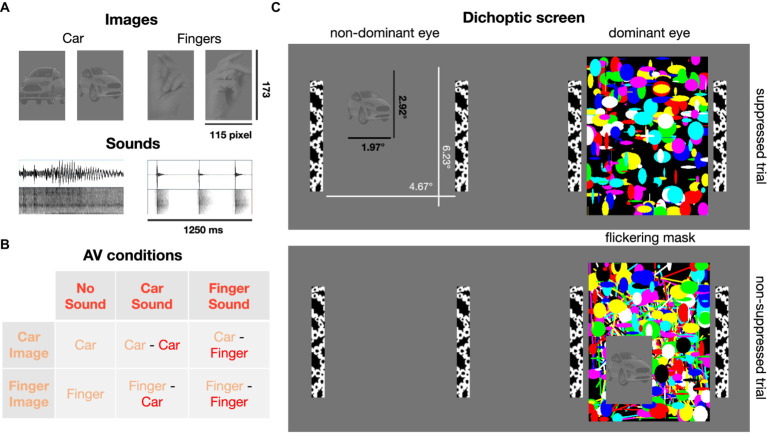
Visual and auditory stimuli. **(A)** Low-contrast images of cars and human hands (2 exemplars shown here, 6 exemplars per image category were used in total) were presented, typically, to the non-dominant eye. Car engine or finger snapping sounds were delivered concurrently with image presentation. **(B)** Six combinations of image and sound categories were tested. **(C)** Dichoptic screen viewed through a stereoscope. A high contrast flickering mask was presented to the dominant eye. Suppression from awareness with continuous flash suppression was achieved by presenting the car or finger image to the other, non-dominant eye (82% of trials). In the non-suppressed control condition (18% of trials), the image was presented on top of the flickering mask and thus clearly visible.

To create mask images, randomly chosen shapes of various colors were overlayed (Figure 1), and, importantly, each set of masks was created anew for each trial. Thus, none of the mask images were ever same, to exclude that systematic mask features influence eye movements. Mask images changed at a frequency of 30 Hz (Sterzer et al., 2008; Alsius and Munhall, 2013).

The screen was divided into two stimulus regions projected to the left and right eye, respectively, by means of the stereoscope. To help fuse the two images, black and white bars framed the stimulus region for each eye (4.67° × 6.23°; Figure 1). Target images were randomly displayed in one of the four quadrants of one stimulus region. In typical suppression trials, the mask was presented to the dominant eye and the low contrast target images were presented to the non-dominant eye to achieve optimal suppression. In the remaining trials, the image was displayed on top of the mask presented to the dominant eye, so that the finger/car image was clearly visible. These non-suppressed, conscious trials were introduced as a positive control, to ensure that participants paid attention and reported the image’s position and content correctly when it was clearly visible. Eye dominance was determined with a hole-in-card test (Miles, 1930) at the beginning of the study for each participant and verified after participants had been familiarized with the experiment (Ding et al., 2018). Six participants exhibited left eye dominance.

Twelve sound files (see Supplementary material, six different sounds of a person snapping their fingers and six different sounds of a starting car engine or an approaching car) were cut to a duration of 1,250 ms and normalized for sound pressure level in Audacity (version 3.2.1). Audio tracks were faded in and out for 25 ms, to avoid abrupt on- and offset. Sounds were delivered diotically through over-ear headphones (Sennheiser HD 418) and sound volume was adjusted to be at a comfortable level for each participant.

**Figure 2:**
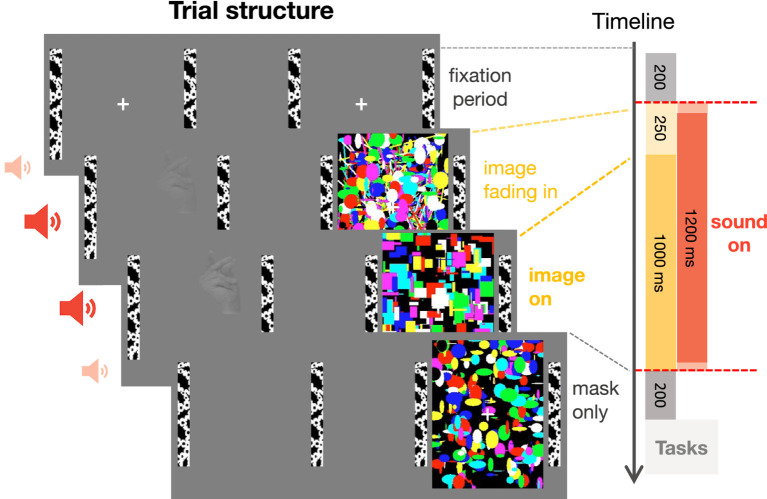
Trial structure. After a mandatory fixation period of 200 ms, an image of a car or a hand was gradually faded in for 250 ms and presented for 1,000 ms to the non-dominant eye while a flickering mask was continuously presented to the dominant eye. Simultaneously to the onset of the visual images, a sound, lasting for the whole duration of image presentation, was presented (except in the no sound condition). The mask was displayed for an additional 200 ms to prevent aftereffects of the target image. After stimulus presentation, participants indicated the location and category of the target image, guessing if the image was successfully suppressed, and rated the visibility of the target image.

Gaze locations of the non-dominant eye were recorded monocularly at 1,000 Hz using an EyeLink 1,000 (SR Research, Ottawa, Canada). To map eye positions onto screen coordinates, 5-point calibration procedures were performed at the beginning of the experiment, before the beginning of a new block, and when participants repeatedly failed to maintain fixation during the initial fixation period of a trial (Figure 2).

Images and sounds were presented via MATLAB (R2019b) using the Cogent toolbox (Wellcome Department of Imaging Neuroscience, University College London).

### Tasks

Participants were instructed to detect any image behind the flickering mask. After each trial, participants answered three questions about the (suppressed) visual stimulus (non-speeded). First, they indicated the quadrant in which they thought the image had been presented (four-alternative forced-choice). They responded by pressing one of four keys on a conventional keyboard. Two ways of quadrant to key assignments were used and counterbalanced across participants. Second, participants indicated the image category (car or fingers, two-alternative forced-choice), again by pressing one of two assigned keys on the keyboard. Third, participants rated the visibility of the target image as either ‘not seen at all,’ ‘brief glimpse,’ ‘almost clear,’ or ‘very clear’ (Sandberg et al., 2010). Each of the three questions was sequentially displayed on the screen. The order of position and categorization tasks was counterbalanced across participants; the visibility rating was always prompted last. The first two tasks served as objective measures of awareness. Participants’ performance allowed us to determine whether participants were able to localize and identify the images above chance level. The visibility rating served as subjective measure of awareness. Participants were instructed to make a guess in the first two tasks if they did not see the target image and to ignore the sounds.

### Procedure

Each trial started with a mandatory fixation period of 200 ms (Figure 2), i.e., the trial only began after participants maintained fixation of a centrally presented fixation cross for at least 200 ms. After the fixation period, the flickering mask started. The target image was gradually faded in for 250 ms, to avoid abrupt image onset and to achieve more effective suppression (Tsuchiya et al., 2006). The sound started directly after the fixation period and lasted for 1,250 ms. The target image was displayed at full contrast for another 1,000 ms before it disappeared. The mask was displayed for an extra 200 ms to prevent afterimages of the target stimulus. There was break every 22 trials (~2.5 min) allowing participants to rest their eyes.

The experiment comprised 528 trials divided into six blocks of 88 trials, administered in two sessions on two separate days. In 432 trials (82% of total trials), the image was presented to the non-dominant eye and the flickering mask to the dominant eye to achieve suppression of the target image from awareness. In 96 trials (18%), the image was displayed on top of the mask presented to the dominant eye, so that the finger/car image was clearly visible. Trial type (suppressed or non-suppressed), image position (upper left, upper right, lower left, lower right), image content (fingers, car), and sound content (fingers, car, no sound) were presented in randomized order.

### Data analysis – behavior

**Figure 3:**
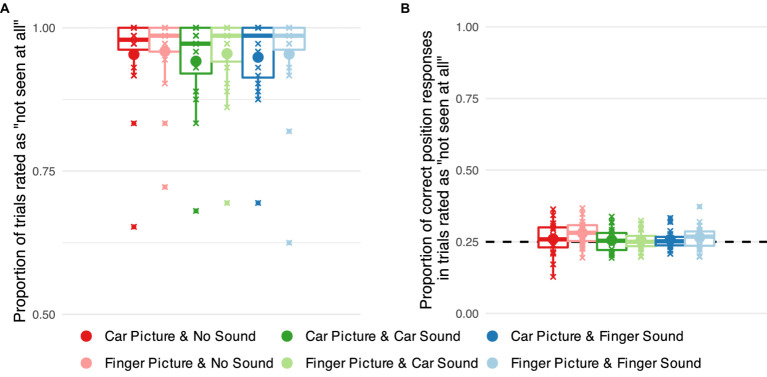
Objective and subjective measures of awareness of target images suppressed from visual awareness using continuous flash suppression. **(A)** Proportion of successfully suppressed trials, i.e., trials rated as ‘not seen at all’ separately for each combination of visual target stimulus category (car vs. finger) and sound category (no sound vs. car sound vs. finger snapping sound). Small markers show subject-level averages, large markers group averages. **(B)** Proportions of correct responses in the position task in successfully suppressed trials for each of the six stimulus categories. The dashed line indicates chance level performance.

The suppressive effects of continuous flash suppression vary inter-and intra-individually (Hesselmann et al., 2016). We employed subjective and objective measures to ensure that only trials in which the image was indeed fully suppressed from awareness were included in the analysis. First, only trials with a subjective visibility rating of zero (‘not seen at all’) were included in the analysis (70 out of 88 trials per condition on average, see Figure 3A for breakthrough rates). Second, to validate these subjective judgments, participants’ average accuracy in the position task was calculated for these subjectively invisible trials. Data from the categorization task were not analyzed further, as an inspection of the results indicated that many participants reported the identity of the sound when the image was successfully suppressed (Supplementary Figure S2).

To analyze whether the success of continuous flash suppression differed across stimulus categories, we recoded the subjective visibility ratings into a binary variable (‘not seen at all’ vs. all other ratings). We conducted a hierarchical logistic regression with predictors image type and sound type, their interaction, and participant-level intercepts to check whether this binary indicator of suppression varied across stimulus categories.

We moreover checked the accuracy of participants’ responses in the localization task in trials for which they indicated to not have seen the target image. We conducted a hierarchical logistic regression on response accuracy with predictors image type and sound type, their interaction, and participant-level intercepts. We additionally compared performance in each category against chance level by calculating planned contrasts against μ=0.25 .

### Data analysis – dwell time

To quantify the effects of the different visual and auditory stimuli on gaze, we analyzed the distribution of fixations across the stimulus region. Trials in which less than 25% of the recorded gaze positions were located inside the stimulus field (for example, because the participant repeatedly blinked during the trial) were excluded from this analysis (<1% of trials).

Gaze positions were transformed into polar coordinates and discretized by dividing the stimulus region into four wedges corresponding to the four quadrants in which a target image could have been displayed. To allow averaging of trials across the four possible target image locations, the coordinates were rotated so that the target image was in the upper-right quadrant. For each target quadrant, we calculated the relative dwell time, i.e., the percentage of time gaze rested in that quadrant relative to the total number of valid eye position samples in the period between 500 ms after image fully on and the end of the trial. To statistically analyze the eyes’ responses to the image, the average percentage of time for which gaze rested in one of the three non-target quadrants was subtracted from the one for the target quadrant, giving a dwell time bias score. A bias score of 0 indicates that a participant looked with equal probability at the target quadrant than at the other three quadrants, a positive score indicates a bias toward looking at the target image even though it was likely suppressed from awareness. A linear mixed model with predictors image type and sound type, their interaction, and participant-level intercepts was fitted to the dwell time bias scores. Bias scores for each condition were contrasted against μ=0 to test for significant biases toward the target image.

## Results

First, we examined the proportion of trials that were rated as ‘not seen.’ When the target image was presented to the non-dominant eye, the vast majority of trials were rated as “not seen” (about 95% of trials on average, see Figure 3A) indicating successful suppression. There was a marginal effect of image category (car vs. finger) on the proportion of trials rated as not seen, $\chi^{2}$ (1) = 3.564, *p* = 0.059, with the car images being rated slightly less often as not seen. No significant effect of sound, $\chi^{2}$ (2) = 2.371, *p* = 0.306 and no significant interaction between image and sound, $\chi^{2}$ (2) = 0.557, *p* = 0.757, emerged.

Second, we analyzed localization accuracy in the position task for trials in which the target image was rated as ‘not seen’ (Figure 3B). The analysis showed no significant effect of image category, $\chi^{2}$ (1) = 0.852, *p* = 0.356, of sound condition, $\chi^{2}$ (2) = 1.591, *p* = 0.452, or their interaction $\chi^{2}$ (2) = 0.599, *p* = 0.742. Planned contrasts against chance level, i.e., 25% correct responses, showed no statistically significant deviations from chance performance in any category.

In sum, our behavioral measures demonstrate that the target images were successfully suppressed from visual awareness in our experimental set-up. That is, in the vast majority of trials, the images were rated as invisible, and participants were unable to localize the images they rated as ‘not seen’.

**Figure 4:**
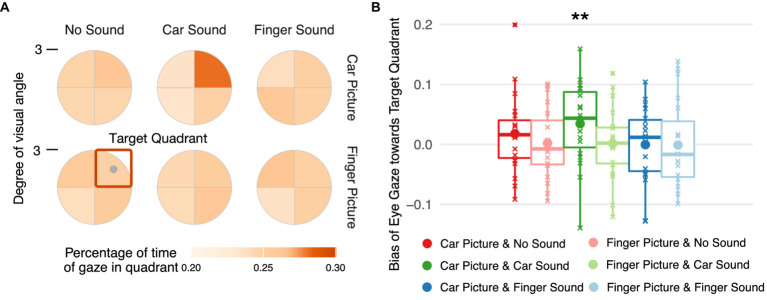
Spatial distribution of eye gaze during stimulus presentation. **(A)** Percentage of time gaze rested in each of the four spatial quadrants during the time interval starting 500 ms after full onset of the target image and ending at trial offset. Gaze coordinates were aligned such that the target center (gray dot) is in the upper right quadrant (orange square). **(B)** Dwell time bias toward the target quadrant per target image and sound condition. The bias in gaze position over time was quantified by subtracting the mean percentage of time the gaze rested in one of the three non-target quadrants from the percentage of time gaze rested on the target quadrant.

Finally, we inspected gaze positions during the stimulation period and statistically analyzed a dwell time bias score reflecting the percentage of time participants’ gaze rested on the target quadrant with respect to the other three quadrants. Only one of the experimental conditions was associated with a significant bias in dwell time (Figure 4A), namely car images paired with car sounds, i.e., the eyes remained longer in the quadrant with a car image than in the other three quadrants when a car sound was played during the image presentation, β=0.032 [0.009, 0.057], *t*(144) = 2.594, *p* = 0.005 (Figure 4B). No significant bias in dwell times emerged for any other condition (see Supplementary Table S1 for all estimated parameters, their 95% highest posterior density intervals, and a regions of practical equivalence analysis confirming the results of the frequentist contrast evaluation).

## Discussion

Threat-related information is prioritized during perceptual processing and guides oculomotor actions even in the absence of awareness (Bannerman et al., 2009; Rothkirch et al., 2012; Vetter et al., 2019). Yet, it remains unclear whether this special link between threat-related information and the oculomotor system is limited to visually conveyed information. In this study, we investigated how sounds influence observers’ eye movements to threat-related images when these images were fully suppressed from visual awareness. Suppression from visual awareness was determined based on subjective visibility ratings and verified for each observer using objective measures. After each trial, participants reported the location and category of the image and rated its visibility on a categorical scale (‘not seen at all,’ ‘brief glimpse,’ ‘almost clear,’ ‘very clear’). We analyzed only those trials in which subjective visibility was zero (Figure 3A), and only data from participants whose objective localization performance across trials with subjectively zero visibility was at chance level (Figure 3B). Critically, across those trials in which images were fully suppressed from visual awareness, we determined using eye tracking how the eyes reacted to threat-related car images compared to non-threat related images of human hands when they were paired with congruent, incongruent, or no sounds. Semantically congruent car sounds indeed guided eye movements toward suppressed threat-related car images even when participants were subjectively and objectively unaware of these images. Images by themselves, i.e., images presented without sounds did not attract the eyes in a significant manner. Thus, image content or low-level visual image features (e.g., contrast or spatial frequency) alone did not guide eye gaze. Also, our observed effect is not a mere audio-visual co-occurrence effect, instead our effect is specific to the combination of threat-related car sounds with car images, driving the eyes closer to car images during full suppression from awareness.

Our findings highlight once more the efficiency of vision and action responding to threats below the threshold of sensory awareness (Yang et al., 2007; Chen et al., 2011; Almeida et al., 2013; Hedger et al., 2015; Bertini et al., 2019). However, in contrast to the majority of previous studies showing threat-related effects with emotional face images (fearful or angry), our study demonstrates that threats conferred by inanimate objects and in different modalities can guide action. The eye gaze effect being stronger for car images when presented concurrently with car sounds resembles the preferential breakthrough of car images in binocular rivalry (with and without sounds, Chen et al., 2011). The ability to quickly detect and respond to potential threats confers a survival advantage (Öhman et al., 2001) and thus might have evolutionary roots. Here we show that the threat associated with an approaching car can guide the eyes toward car images even in the entire absence of visual awareness. Indeed, in modern real-life situations, cars and car sounds are often linked to potential danger (Bradley and Lang, 2017), triggering heightened attention and rapid, automatic responses. Hence, the here revealed link between the threat posed by cars and oculomotor actions might suggest an adaption of an evolutionary rooted brain mechanism to modern-day threats.

Furthermore, our results suggest cross-modal enhancement of threat-related images suppressed from visual awareness. Gaze exclusively rested more frequently on “unseen” car images when congruent car sounds were displayed. Previously, we found that threat-related emotional faces (without sounds) guided eye gaze in the absence of awareness (Vetter et al., 2019). Here, we extended these findings to threat-related objects in a multisensory context, indicating cross-modal facilitation of eye gaze guidance in the absence of awareness. In turn, this extends the range of effects of cross-modal facilitation of suppressed visual information from explicit perceptual reports to the unaware guidance of oculomotor actions. Previous studies reported that congruent auditory (Chen and Spence, 2010; Chen et al., 2011; Alsius and Munhall, 2013; Lunghi et al., 2014; Lee et al., 2015; Delong and Noppeney, 2021) and tactile (Lunghi et al., 2010; Hense et al., 2019; Liaw et al., 2022) information facilitates the breakthrough of suppressed visual information into awareness. Here, we find the same enhancing effect of congruent auditory information on suppressed visual information, however, the effect shows in unaware eye movements, and thus, unaware actions.

This unaware multisensory effect on eye gaze could be supported by the fast subcortical retinocollicular pathway (Spering and Carrasco, 2015). The superior colliculus (SC) receives inputs from the retinal ganglion cells and the auditory cortex (Benavidez et al., 2021; Liu et al., 2022), and may contribute to gaze changes toward car images in response to sounds. SC is known for its multimodal function in sensory integration that facilitates neural and oculomotor responses. Multisensory integration effects are strongest when weak unisensory stimuli are combined (Meredith and Stein, 1986), especially when recording from SC neurons (Stein et al., 2020). It is thus likely that in our study the congruent sounds elicited heightened representations of the suppressed, low contrast car image not only in visual areas but also in the SC, and thus facilitated eye movements. That this crossmodal effect is specific to threat-related stimuli, in our case the car, could be accounted for by the SC-pulvinar-amygdala circuit (LeDoux, 1998; Rafal et al., 2015; Kragel et al., 2021; Kang et al., 2022). For example, activation of the colliculo-amygdalar circuitry is important for rodent freezing behavior following fear-evoking visually approaching stimuli (Wei et al., 2015). The amygdala is also closely linked to the inferior colliculus (the acoustic relay center) via the thalamus (LeDoux et al., 1990). As sounds increase in intensity, thus conveying warning, the amygdala is activated alongside the posterior temporal sulcus within the auditory cortex (Bach et al., 2008). Therefore, the observed sound effects on eye gaze to suppressed car images could potentially be explained by a coordinated subcortical system involving the colliculi and amygdala (Jiang and He, 2006; Méndez-Bértolo et al., 2016; Wang et al., 2023). In addition to these subcortical structures, early sensory cortices of all sensory modalities are involved in threat processing (Li and Keil, 2023). For example, auditory cortex encodes the emotional valence of sounds during threat assessment (Concina et al., 2019). Thus, in addition to processing along the subcortical route, visual, auditory and multisensory cortices might have played an important role in processing threat-related stimulus content in the absence of awareness.

In the present study, we focused on the comparison of eye responses to suppressed threat-related car and neutral hand images and sounds. Future studies could test the generability of our findings to other types of threat-related naturalistic stimuli, e.g., using standardized databases of affective stimuli (e.g., Balsamo et al., 2020). Also, due to a general positivity bias (e.g., Fairfield et al., 2022) and given that attention is biased toward positive natural sounds (e.g., Wang et al., 2022), future studies could test how positively valenced stimuli may modulate eye movements in the absence of visual awareness.

## Conclusion

Our results show that threat conferred by car images and engine sounds attracts eye gaze even in the entire absence of visual awareness. Thus, multisensory enhancement of threat-related unaware visual representations leads to specific actions, potentially mediated by subcortical brain circuits responsible for eye movement control and multisensory interaction.

## Data availability statement

The raw data supporting the conclusions of this article will be made available by the authors, without undue reservation.

## Ethics statement

The studies involving humans were approved by Psychology Ethics Committee, University of Fribourg. The studies were conducted in accordance with the local legislation and institutional requirements. The participants provided their written informed consent to participate in this study.

## Author contributions

JH: Conceptualization, Data curation, Formal analysis, Investigation, Visualization, Writing – original draft, Writing – review & editing. SB: Conceptualization, Data curation, Formal analysis, Supervision, Visualization, Writing – original draft, Writing – review & editing. PV: Conceptualization, Funding acquisition, Supervision, Writing – original draft, Writing – review & editing.
