# Supplementary material for: Auditory guidance of eye movements toward threat-related images in the absence of visual awareness
Source: Front Hum Neurosci. 2024 Aug 8;18:1441915. doi: 10.3389/fnhum.2024.1441915 (PMC11338778; doi:10.3389/fnhum.2024.1441915)
Supplement: Supplementary file 1 [file Data_Sheet_1.PDF]

frontiers

# *Supplementary Material*

## Auditory guidance of eye movements towards threat-related images in the absence of visual awareness

Authors: Junchao Hu[1], Stephanie Badde[2*] & Petra Vetter[1*]

[1]Visual & Cognitive Neuroscience Lab, Dept. of Psychology, University of Fribourg, Switzerland.

[2]Dept. of Psychology, Tufts University, USA.

* equal contribution

**Correspondence:** Corresponding Author: petra.vetter@unifr.ch

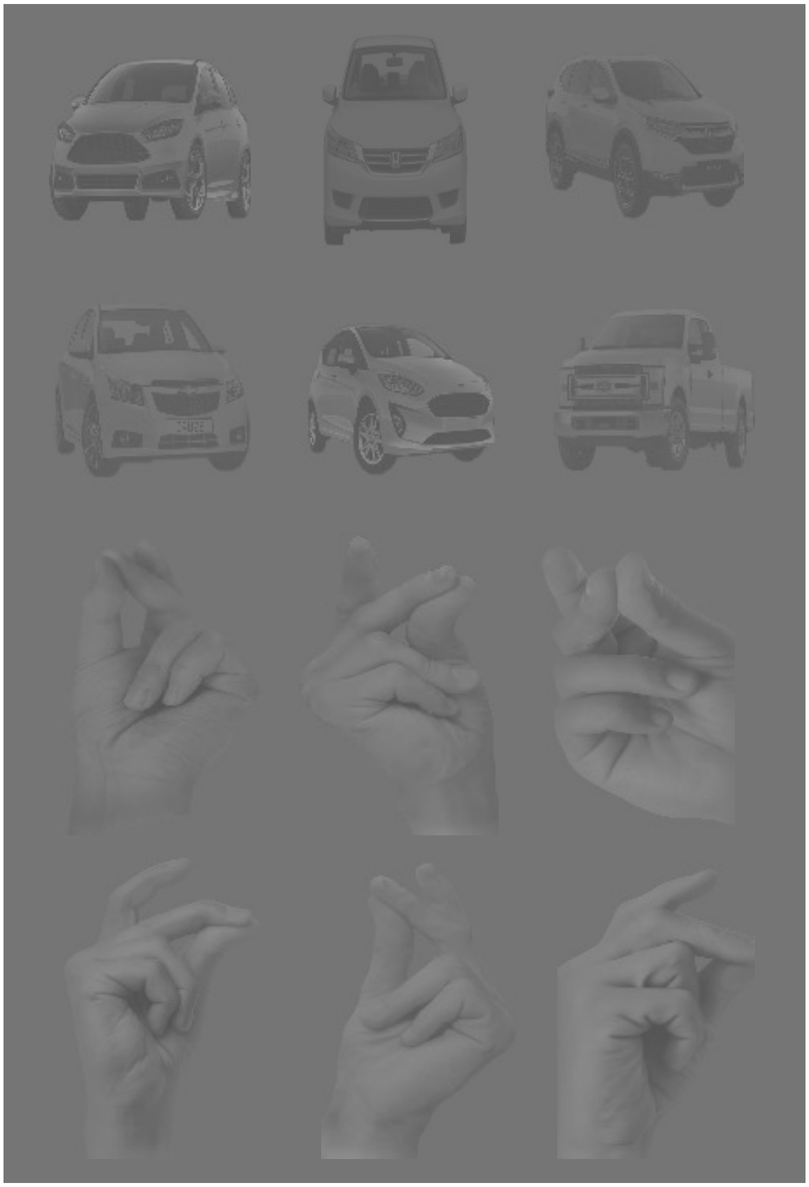

**Supplementary Figure 1.** Car and finger images as used in the experiment.

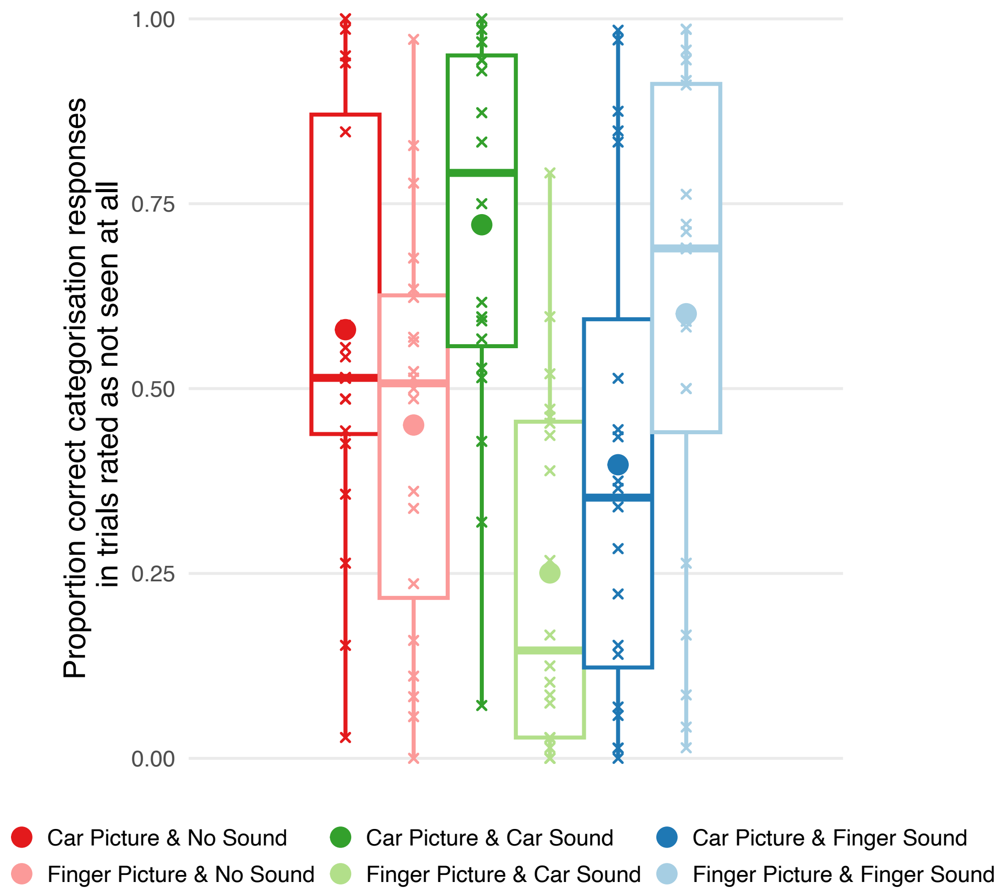


**Supplementary Figure 2.** Second objective measure of awareness of car and finger images masked from continuous flash suppression per sound condition. This figure shows the proportion of correct responses for the categorization task ("Was it a car or finger?") in successfully suppressed trials, i.e., trials rated as not seen at all. Chance level at correctly categorizing the image content is 50%.

**Supplementary Table 1.** Dwell time bias score of each condition contrasted against $\mu = 0$.

| Image | Sound | Estimate and 95% HDI | Statistical test of the contrast | Percentage of MCMC samples in ROPE |
|---|---|---|---|---|
| **Car Picture** | **Car Sound** | $\boldsymbol{\beta = 0.032}$ **[0.009, 0.057]** | $\boldsymbol{t(144) = 2.594}$, $\boldsymbol{p = 0.005}$ | **1.5%** |
| Finger Picture | Car Sound | $\beta = 0.003$ [-0.023, 0.027] | $t(144) = 0.222$, $p = 0.412$ | 59.34% |
| Car Picture | Finger Sound | $\beta = 0.005$ [-0.019, 0.031] | $t(144) = 0.385$, $p = 0.351$ | 55.03% |
| Finger Picture | Finger Sound | $\beta = -0.003$ [-0.028, 0.023] | $t(144) = -0.256$, $p = 0.601$ | 57.89% |

| | | | | |
|---|---|---|---|---|
| Car Picture | No Sound | $\beta = 0.012$ [-0.012, 0.037] | $t(144) = 0.936$, $p = 0.175$ | 42.76% |
| Finger Picture | No Sound | $\beta = 0.003$ [-0.022, 0.029] | $t(144) = 0.237$, $p = 0.407$ | 58.71% |

Note: MCMC samples were generated using Stan via brm(). The region of practical equivalence (ROPE) ranges from -0.01 to 0.01.
